# New Technologies for Glaucoma Imaging

**DOI:** 10.1155/2011/608975

**Published:** 2011-09-21

**Authors:** Christopher K. S. Leung, Felipe A. Medeiros, David Garway-Heath, David S. Greenfield, Robert N. Weinreb

**Affiliations:** ^1^Department of Ophthalmology and Visual Sciences, The Chinese University of Hong Kong, Kowloon, Hong Kong; ^2^Hamilton Glaucoma Center, University of California, San Diego, La Jolla, CA 92093, USA; ^3^Moorfields Eye Hospital, London, UK; ^4^Bascom Palmer Eye Institute, University of Miami Miller School of Medicine, FL, USA; ^5^Shiley Eye Center, University of California, San Diego, La Jolla, CA 92093, USA

The evolution of imaging technologies over the past decade has reshaped our landscape in diagnosing and managing patients with glaucoma. Adopting technological advances and translating new information into clinical practice pose new challenges to both clinicians and research scientists. This special issue includes five papers covering some of the recent advances in ocular imaging and electrophysiology technologies and demonstrates how such advancement augments our understanding of glaucoma. 

Since its introduction in 2005, the spectral-domain optical coherence tomography (SD-OCT) has received considerable attention for retinal nerve fiber layer (RNFL) and optic disc imaging. With improved axial resolution to discern individual retinal layers, new parameters such as the ganglion cell complex (GCC) and the photoreceptor layer thicknesses can now be measured reliably with SD-OCT. S. T. Takagi and et al. demonstrate that macular GCC thickness could serve as a biomarker for early detection of glaucoma. With a high density of retinal ganglion cells in the macula, it is conceivable that measuring macular GCC thickness would be valuable for glaucoma assessment. By contrast, the outer retina would be expected to be unaffected. Unexpectedly, N. Fan et al. show that the outer nuclear layer is thicker in mild glaucomatous eyes than in normal eyes. This finding suggests glaucomatous damage may involve structural change in the photoreceptors.

Full-field electroretinogram (ERG) has been considered less useful for glaucoma evaluation. S. Machida et al. show that a photopic negative response (PhNR) (a negative deflection following the photopic b-wave) obtained from a focal ERG system could indicate functional abnormality of retinal ganglion cells and has a high diagnostic performance to detect early glaucoma. While the potential of focal ERG PhNR for objective functional assessment appears promising, prospective longitudinal studies are needed to validate its use in glaucoma patients. 

Intraocular pressure (IOP) is a major risk factor for glaucoma progression. Yet, it has long been recognized that there are factors other than IOP that could modify the course of the disease. In a randomized double-masked study comparing dorzolamide/timolol and latanoprost/timolol fixed combination for treatment in glaucoma patients, I. Janulevičiene et al. report that a number of hemodynamic parameters including blood pressure, ocular perfusion pressure, and ophthalmic and central retinal artery vascular resistance are associated with RNFL and/or visual field progression independent of IOP reduction. Although this study is limited by a small sample size (15 patients in each arm) and a high progression rate (40% and 47% of patients progressed, resp.), the result could stimulate more research investigating the roles of ocular and systemic hemodynamics in glaucoma progression. 

Multiphoton microscopy is a relatively new modality for ophthalmic imaging. E. A. Gibson et al. summarize the applications of multiphoton microscopy for retinal and transscleral imaging and demonstrate the feasibility to visualize the collagen fibers in human trabecular meshwork *ex vivo*. The exciting development of multiphoton imaging will undoubtedly provide new data for potential application in the clinic in the near future. 

The improvement in patient care depends upon continued research and development of new tools and new techniques to unfold the pathobiology of glaucoma for early diagnosis and treatment. It is sufficient to say that glaucoma imaging forms one of the cornerstones in the advancement of glaucoma care.

*Christopher K. S. Leung* *Christopher K. S. Leung* *Felipe A. Medeiros* *Felipe A. Medeiros* *David Garway-Heath* *David Garway-Heath* *David S. Greenfield* *David S. Greenfield* *Robert N. Weinreb* *Robert N. Weinreb*

